# Frailty as a predictor of mortality and readmission rate in secondary mitral regurgitation

**DOI:** 10.1007/s00508-022-02138-4

**Published:** 2023-01-12

**Authors:** Robert Uzel, Richard Rezar, Raphael Romano Bruno, Sarah Wernly, Christian Jung, Georg Delle Karth, Christian Datz, Friedrich Hoppichler, Bernhard Wernly

**Affiliations:** 1 Department of Internal Medicine, Saint John of God Hospital, Teaching Hospital of the Paracelsus Medical Private University, Kajetanerplatz 1, 5020 Salzburg, Austria; 2 Department of Cardiology, Klinik Floridsdorf, Brünner Str. 68, 1210 Vienna, Austria; 3 Department of Cardiology and Intensive Care Medicine, Paracelsus Medical University of Salzburg, Müllner Hauptstr. 48, 5020 Salzburg, Austria; 4 Department of Cardiology, Pulmonology and Vascular Medicine, Medical Faculty, Heinrich-Heine-University Duesseldorf, Moorenstr. 5, 40225 Duesseldorf, Germany; 5 Department of Internal Medicine, General Hospital Oberndorf, Teaching Hospital of the Paracelsus Medical Private University, Paracelsusstr. 37, 5110 Oberndorf, Austria; 6 Special Institute for Preventive Cardiology and Nutrition, SIPCAN—Initiative für ein gesundes Leben, Salzburg, Austria; 7 Institute of general practice, family medicine and preventive medicine, Paracelsus Medical University, Strubergasse 21, 5020 Salzburg, Austria

**Keywords:** Mitral valve insufficiency, Conservative treatment, Prognosis, Heart failure, Decision making

## Abstract

**Introduction:**

Selection in patients with functional mitral regurgitation (MR) to identify responders to interventions is challenging. In these patients, frailty might be used as a multidimensional parameter to summarize the resilience to stressors. Our objective was to evaluate frailty as a predictor of outcome in patients with moderate to severe secondary MR.

**Methods:**

We conducted a single-center retrospective observational cohort study and included 239 patients with moderate to severe secondary MR aged 65 years or older between 2014 and 2020. Echocardiography was performed at baseline; frailty was evaluated using the clinical frailty scale (CFS). The combined primary endpoint was hospitalization for heart failure and all-cause mortality.

**Results:**

A total of 53% (127) of all patients were classified as CFS 4 (living with mild frailty) or higher. Frail patients had a higher risk for the combined endpoint (hazard ratio, HR 3.70, 95% confidence interval, CI 2.12–6.47; *p* < 0.001), 1‑year mortality (HR 5.94, 95% CI 1.76–20.08; *p* < 0.001) even after adjustment for EuroSCORE2. The CFS was predictive for the combined endpoint (AUC 0.69, 95% CI 0.62–0.75) and outperformed EuroSCORE2 (AUC 0.54, 95% CI 0.46–0.62; *p* = 0.01). In sensitivity analyses, we found that frailty was associated with adverse outcomes at least in trend in all subgroups.

**Conclusion:**

For older, medically treated patients with moderate to severe secondary mitral regurgitation, frailty is an independent predictor for the occurrence of death and heart failure-related readmission within 1 year and outperformed the EuroSCORE2. Frailty should be assessed routinely in patients with heart failure to guide clinical decision making for mitral valve interventions or conservative treatment.

## Introduction

Valvular heart disease (VHD) affects 50% of the population aged 65 years and older, with a general increase in age and higher prevalence in men and is also a common reason for heart failure (HF) in this population [1]. Given increasing life expectancy, the number of patients with clinically significant VHD is expected to double by 2050 [1, 2]. From a socioeconomic perspective, HF is the most common reason for hospitalization in Germany and will continue to be a central public health issue in an aging population [3]. Mitral regurgitation (MR) is the second most common valvular heart disease [4]. While primary MR is a mechanical problem of the valve itself, secondary MR mostly results from geometrical distortion, which is usually caused by a left-ventricular pathology [5]. According to the European Society of Cardiology (ESC) guidelines, optimal medical therapy (OMT) is the first line treatment strategy, followed by cardiac resynchronization therapy (CRT) in appropriate cases, which can lead to increasing closing forces and resynchronization of papillary muscles with short-term and long-term improvement of MR [6–9]. Mitral valve intervention should be considered if symptoms persist [10]; however, treatment and prognostication of patients with secondary MR is difficult in clinical practice. OMT is underutilized and the rate of surgery, when indicated, is low, often due to poor general health status, comorbidities, advanced age, and frailty [5, 11, 12]. As with VHD, the increasing numbers of frail patients poses a serious problem for healthcare systems especially in western countries with aging populations. Its prevalence increases with age and is approximately 10% in community-dwelling adults aged 65 years and older. In the inpatient setting, this number mounts up to a range of 25–80% [13–16]. For frail patients, the risk of an unfavorable outcome and persistent impairment of quality of life (QoL) after severe illness has been demonstrated in multiple publications, as recently for COVID-19 infection [17, 18]. A well-established frailty assessment tool is the judgement-based CFS, which can be used to summarize the overall level of fitness and provides a prediction of short-term outcome [19, 20].

The aim of our study was to evaluate frailty as an outcome predictor in a real-world population with moderate to severe secondary MR to support clinical decision making for mitral valve intervention.

## Patients, material and methods

We conducted a single-center retrospective observational cohort study from 1 January 2014–30 september 2020. The study was approved by the local ethics committee (1170/2020). Our hospital electronic health care records were searched for patient cases with ICD-10 codes related to mitral regurgitation (MR). Complete clinical, echocardiographic and pharmacologic data were obtained from the digital fever chart by means of comprehensive chart review. Only adults aged 65 years and older were analyzed. Further inclusion criteria were any-cause hospitalization and in-hospital performed echocardiography with a diagnosis of moderate to severe MR. Individuals with primary MR and patients who were referred to surgery or mitral valve repair were excluded. The diagnosis primary or secondary MR was made according to European Society of Cardiology guidelines for echocardiography [21]. All patients underwent 3D echocardiography assessed by experienced echocardiographers at baseline.

**Fig. 1:**
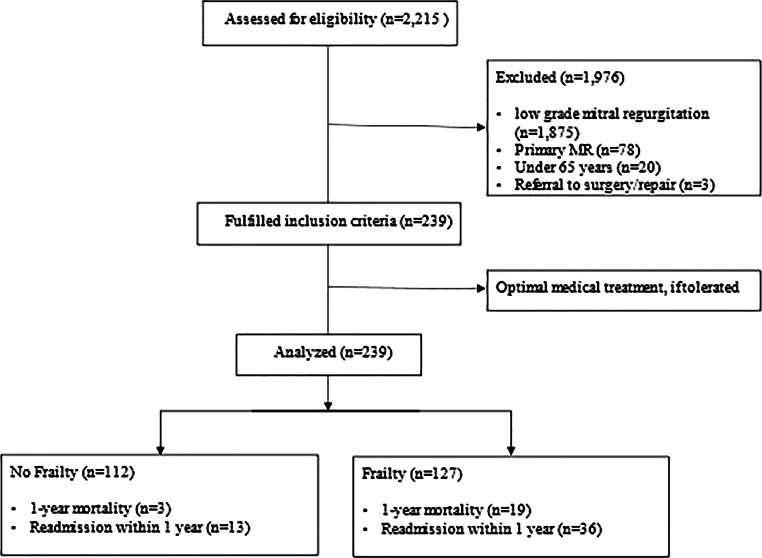
Flow chart of patient inclusion

The combined primary endpoint was hospitalization due to heart failure and all-cause death. To reduce confounding effects due to competing risks, we decided to shorten the observation period to a follow-up of 12 months. Frailty was assessed from the nursing records using the revised version (2.0) of the CFS [19]. As a binary variable, patients with a score of 4 or higher were considered as frail since the former level 4 “vulnerable” is now “living with mild frailty”. This reflects the increased risk with the corresponding degree of deficit accumulation [22]. As a categorical variable, we additionally differentiated frail patients into two groups: mild to moderate (CFS 4–6) and severe frailty (CFS ≥ 7), according to recent publications [23]. For the assessment of general cardiovascular risk, we used the long-established EuroSCORE II. Poor mobility as a result of musculoskeletal or neurological dysfunction was assessed from the nursing records. A total of 2215 patient cases were screened, 239 met inclusion criteria and were analyzed (shown in Fig. 1). Patients were either found to be ineligible for surgery because of absent medical indications, comorbidities, frailty, severe dementia, life-threatening malignancy, other clinical reasons or at their own request.

**Fig. 2:**
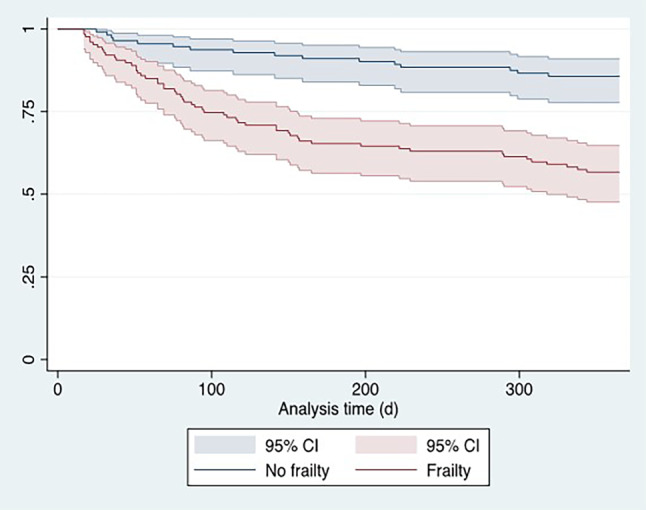
Kaplan-Meier curve illustrating survival dependent on clinical frailty scale: no frailty (CFS 1–3), frailty (CFS 4–9). *Y‑axis *depicts survival probability. *CFS* clinical frailty scale*, CI* confidence interval

**Fig. 3:**
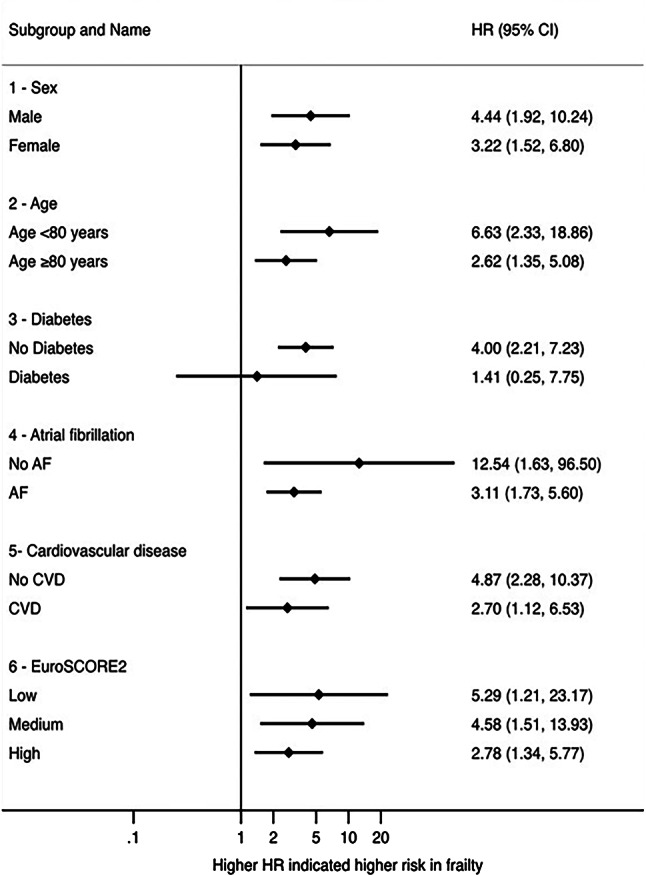
Forest plot of frailty as a binary variable for different subgroups. *AF* atrial fibrillation,* CI* confidence interval, *CVD* cardiovascular disease, *HR* hazard ratio

Baseline characteristics were expressed as median with interquartile range (IQR) for continuous variables in parentheses. Differences between groups were calculated using U‑test. Categorial variables, were expressed as frequencies and percentages, and χ^2^-test was used to calculate differences between groups. Survival and hospitalization were visualized by means of Kaplan Meier plots in Fig. 2. We fitted univariate and multivariable Cox regression proportional hazard models, with the combined endpoint or 1‑year mortality as dependent variable, frailty as categorical or binary variable and the covariable EuroSCORE2 for the multivariable model as fixed effects. We obtained hazard ratios (HR) and adjusted HR (aHR) with respective 95% confidence intervals (CI). We plotted the univariable HR of the model with the combined endpoint as dependent variable and frailty as binary variable as independent variable in Fig. 3. We chose the covariable EuroSCORE2 based on our own clinical experience and previous literature [24, 25]. HR describes the change in risk of the respective dependent variable (combined endpoint or 1‑year mortality) for one specific category versus a reference category for categorical variables. A HR > 1 suggests an increase in the risk of death, HR < 1 suggests a decrease in the risk of death. All tests were two-sided, and a *p*-value of < 0.05 was considered statistically significant. As not all parameters were available for all categories, patients had to be excluded for the subgroup analyses. For this reason, not all patient numbers add up to 100% (see tables). Stata 17 was used for all statistical computations (Stata Statistical Software: Release 17. StataCorp LLC, College Station, TX, USA).

## Results

A total of 239 patients with moderate to severe mitral regurgitation were included in the final analysis. All patients received OMT, if tolerated. Table 1 displays the baseline characteristics of patients dichotomized in no frailty (CFS 1–3) versus frailty (CFS ≥ 4). The prevalence of frailty was 53% for the whole cohort. There was no difference in reason for hospitalization between the two groups. Patients were predominantly of female sex (56% vs. 58%; *p* = 0.75), frail patients were older (84 ± 7 vs. 79 ± 8; years *p* < 0.001). Echocardiographic parameters like left ventricular ejection fraction, left atrial size and the presence of aortic stenosis did not differ significantly (see Table 1).

**Table 1:** Baseline characteristics of the study population. Continuous variables are given as median (interquartile ranges in parentheses). Categorial variables are given as percentages and frequencies in parentheses. LVEF and renal function cut-offs were chosen according to EuroScore2 cut-offs

	No frailty (*n* = 112)	Frailty (*n* = 127)	*p*-value
*Age—years*	80 (74–85)	85 (80–89)	< 0.001
*Male sex*	44% (49)	42% (53)	0.75
*Reason for hospitalization*	–	–	0.912
Heart failure	50% (55)	50% (64)	–
Other cardiologic diseases	14% (15)	12% (15)	–
Other causes	37% (41)	38% (49)	–
**Echocardiography**			
*Mitral regurgitation*	–	–	0.45
Moderate	71% (80)	67% (85)	–
Severe	29% (32)	33% (42)	–
*Vena contracta—mm*	6 (5–7)	6 (5–8)	0.39
*LVEDD—mm*	49 (42,5–57)	50 (44–59)	0.45
*Left atrial size—cm*^*2*^	28 (23–35)	30 (25–35)	0.61
*LVEF*	–	–	0.61
Preserved (≥ 50%)	46% (51)	43% (54)	–
Mildly reduced (41–49%)	25% (28)	22% (28)	–
Reduced (≤ 40%)	29% (33)	35% (45)	–
*Aortic stenosis*	–	–	0.98
Absent	81% (91)	79% (100)	–
Mild	5% (6)	6% (8)	–
Moderate	6% (7)	6% (8)	–
Severe	7% (8)	8% (10)	–
**Comorbidities**			
*EuroSCORE2*	5.9% (6.4%)	6.8% (8.5%)	0.37
*Coronary artery disease*	33% (37)	22% (28)	0.057
*Diabetes mellitus*	17% (19)	24% (30)	0.20
*Insulin-dependent*	4% (4)	6% (8)	0.34
*Atrial fibrillation*	71% (79)	70% (89)	0.94
*Extracardiac arteriopathy*	22% (25)	35% (44)	0.036
*Previous cardiac surgery*	5% (6)	6% (7)	0.96
*Chronic lung disease*	16% (18)	15% (19)	0.81
*NYHA classification*	–	–	0.57
I	45% (50)	43% (55)	–
II	14% (16)	15% (19)	–
III	26% (29)	20% (26)	–
IV	15% (17)	21% (27)	–
*CCS class 4 angina*	5% (6)	6% (7)	0.96
*Recent myocardial infarction—within 90 days*	2% (2)	0% (0)	0.13
*Renal function (GFR)*	–	–	0.37
Normal (> 85 ml/min)	22% (25)	17% (21)	–
Moderately impaired (50–85 ml/min)	54% (60)	53% (67)	–
Severely impaired (< 50 ml/min)	24% (27)	31% (39)	–

*CCS* Canadian Cardiovascular Society, *GFR* glomerular filtration rate, *LVEDD* left ventricular end-diastolic diameter, *LVEF* left ventricular ejection fraction, *PISA* proximal isovelocity surface area

Table 2 presents characteristics of geriatric assessment. Frail patients suffered more often from dementia (28% vs. 5%; *p* < 0.001) and poor mobility (65% vs. 21%; *p* < 0.001). They required higher levels of assistance, whether from family members, home care or nursing homes (17% vs. 3%; *p* < 0.001). The rate of polypharmacy was high in both groups (83% vs. 93%; *p* < 0.018).

**Table 2:** Characteristics of the geriatric assessment. Categorial variables are given as percentages and frequencies in parentheses

	No frailty (*n* = 112)	Frailty (*n* = 127)	*p*-value
*Dementia*	5% (6)	28% (35)	< 0.001
*Poor mobility*	21% (23)	65% (83)	< 0.001
*Polypharmacy—≥* *5 medications daily*	83% (93)	93% (118)	0.018
*Frailty (CFS)*	–	–	< 0.001
Non-frail (1–3)	100% (112)	0% (0)	–
Mild to moderate (4–6)	0% (0)	70% (89)	–
Severe (7–9)	0% (0)	30% (38)	–
*Living situation at admission*	–	–	< 0.001
Autonomous	78% (87)	37% (47)	–
Support from relatives	14% (16)	27% (34)	–
Home nursing care	5% (6)	19% (24)	–
Nursing home	3% (3)	17% (22)	–

*CFS* Clinical Frailty Scale

Frailty was significantly associated with the occurrence of 1‑year mortality (3% vs. 15%; *p* = 0.001) as well as the combined endpoint at 1 year (14% vs. 43%; *p* < 0.001) (see Table 3).

**Table 3:** Occurrence of the primary endpoints. Categorial variables are given as frequencies and percentages in parantheses

	No frailty (*n* = 112)	Frailty (*n* = 127)	*p*-value
*1‑year mortality*	3% (3)	15% (19)	0.001
*Combined endpoint 1‑year*	14% (16)	43% (55)	< 0.001

Fig. 2 shows the survival of frail versus non-frail patients using a Kaplan-Meier curve. Frail patients evidenced a higher risk for the combined endpoint (HR 3.70, 95% CI 2.12–6.47; *p* < 0.001) as well as 1‑year mortality (HR 5.94, 95% CI 1.76–20.08; *p* < 0.001). Frailty remained associated with higher risk for the combined endpoint (aHR 3.69, 95% CI 2.11–6.44; *p* < 0.001) and 1‑year mortality (aHR 6.02, 95% CI 1.78–20.35; *p* = 0.004) after adjustment for EuroSCORE2. The CFS was predictive for the combined endpoint (AUC 0.69, 95% CI 0.62–0.75) and outperformed EuroSCORE2 (AUC 0.54, 95% CI 0.46–0.62; *p* = 0.01).

Further, the CFS predicted 1‑year mortality (AUC 0.67, 95% CI 0.58–0.77) better than EuroSCORE2 (AUC 0.49, 95% CI 0.36–0.62; *p* = 0.03). The categorical frailty scale was associated with the combined endpoint in univariate and multivariable modelling (see Table 4).

**Table 4:** Univariate and multivariable Cox regression proportion hazard model

	Univariate model			Multivariable model		
	HR	95% CI	*p*-value	aHR	95% CI	*p*-value
*No frailty*	Reference	–	–	Reference	–	–
*CFS 4–6*	3.39	1.88–6.11	< 0.001	3.35	1.85–6.05	< 0.001
*CFS* *>* *6*	4.49	2.31–8.74	< 0.001	4.54	2.33–8.86	< 0.001

*aHR* adjusted hazard ratio,* CFS* clinical frailty scale,* CI* confidence interval, *HR* hazard ratio

In sensitivity analyses, we found that frailty was associated with adverse outcomes at least in trend in all subgroups (shown in Fig. 3).

## Discussion

Acute heart failure as a result of secondary mitral regurgitation is one of the most common reasons for admission to internal medicine or cardiology departments. The prevalence of frailty increases with age and ranges about 10% in community-dwelling adults aged 65 years and older [13]. In the inpatient setting, this range increases up to values of 25–80%, depending on which institution is examined [14–16]. Therefore, the data analyzed here are representative for European hospitals and the patients cared for. From the perspective of a practice-oriented approach, this study aimed to evaluate frailty as a predictor of outcome in patients with moderate to severe secondary MR to support clinical decision making for mitral valve intervention or conservative treatment.

The major findings of the present study were: 1) frail patients had a significantly higher rate of reaching the combined primary endpoint (death and readmission due to heart failure). 2) In multivariable analysis, frailty was an independent predictor of the occurrence of the primary endpoints. 3) For that purpose, the CFS was a better prognostic tool than the EuroSCORE II. 4) Overall mortality was low in both groups.

Metze et al. showed that frail patients undergoing percutaneous mitral valve repair face a twofold increase in hazard of death or heart failure-related readmission in long-term outcomes compared to nonfrail patients. Frailty as assessed using the Fried criteria is in this study an independent predictor of adverse outcomes after adjustment for logistic EuroSCORE [26]. Our study extends these findings to conservatively treated patients with secondary MR. A standardized frailty assessment seems to apply for both conservative and interventional treatment.

According to expectations, mortality as well as the readmission rate are both significantly higher in the frailty group; however, it has to be mentioned that despite numerous readmissions, overall mortality is still low in both groups. These findings are consistent with previous trials in patients with conservatively and interventionally treated secondary MR [27]. Nevertheless, the treatment of symptomatic secondary MR is characterized by an under-use of medical and surgical treatment [12]. The most frequent characteristics for withheld surgery are impaired LVEF, older age, and comorbidities [11]. The high readmission rates in combination with low mortality and the improvable treatment show that further treatment in this patient group is reasonable in order to avoid a self-fulfilling prophecy. This is supported by the fact that we found a frailty rate of 53% (127) in our patients (only three were excluded before because of surgical referral), which is remarkably high compared to other nationwide samples or study populations that contained mostly younger patients with fewer comorbidities [27, 28]. Given that, one of the main strengths of our study is its real-world character and the high quality of data collection and selection.

Recent findings show that other predictive variables for increased mortality, regardless of intervention, are older age, high Charlson comorbidity index, renal dysfunction, anemia, elevated right atrial pressure (estimated by echocardiography), admission for heart failure, and lack of mitral valve intervention [29, 30]. In our sensitivity analysis, frailty as assessed by the CFS, was associated with adverse outcomes at least in trend in almost all of the parameters mentioned above and should therefore be adopted in evaluation of patients with secondary MR to support treatment decisions.

Furthermore, in univariate analysis, CFS resulted in a higher AUC compared to EuroSCORE2. The poorer performance of the EuroSCORE II might be seen in the light of the fact that this score was designed for the eligibility of cardiac surgery in mainly younger patients. Nevertheless, there does not seem to be a more appropriate risk score for outcome prediction in this sample. According to recent studies, our findings strengthen the presumption that the clinical judgement of patients by means of CFS is a better evaluation tool than the measurement of various clinical parameters and assessment of comorbidities and also predicts the outcome [31]. This supports the well-established practice, in which the application of complex scores is largely impracticable and physicians base their decisions on clinical presentation, which reflects the patients physiological reserve [32]. Therefore, the CFS as a multidimensional parameter can be used as a prognostic marker for the outcome of patients with moderate to severe secondary MR.

### Study limitations

An important limitation is that this is a single-center study with a small cohort of patients, which reflects common patients in non-tertiary care centers. The inclusion was not focused on candidacy for intervention.

Furthermore, all variables were obtained retrospectively and frailty was assessed using nursing documentation. There might be a selection bias, given the fact that patients were recruited via ICD-10 diagnoses only. The recording of every single diagnosis in our digital fever chart is conducted by doctors in charge; however, the accuracy of these recordings and the consecutive completeness of our data cannot be guaranteed. For adjustment, we used EuroSCORE2, in awareness of the fact that this score is not validated in this setting. Nevertheless, EuroSCORE2 is routinely assessed and commonly used for cardiac risk evaluation as it has a high degree of familiarity.

No objective frailty assessment method was used, e.g. 5‑minute walk test, grip strength, etc. Frailty was a subjective determination using the CFS. No additional frailty assessments were used, as the CFS is reliable and comparable to more complex methods, such as Frieds frailty phenotype in identifying frailty [33]. We did not re-evaluate frailty during follow-up. Furthermore, the CFS is not qualified to differentiate between transient or chronic illnesses [18].

## Conclusion

For older, medically treated patients with moderate to severe secondary mitral regurgitation, frailty is an independent predictor for the occurrence of death and heart failure-related readmission within 1 year. In univariate analysis, CFS resulted in a higher AUC compared to EuroSCORE2. We therefore believe that at least in an older medically treated population, reflected in our study cohort, CFS is more useful than EuroSCORE2. This is supported by our own clinical experience that CFS is easier to implement in everyday clinical practice compared to EuroSCORE2. CFS should therefore be assessed routinely in patients with heart failure to guide clinical decision making for mitral valve interventions or conservative treatment.
